# Climate Change and Human Disturbance Can Lead to Local Extinction of Alpine Rock Ptarmigan: New Insight from the Western Italian Alps

**DOI:** 10.1371/journal.pone.0081598

**Published:** 2013-11-19

**Authors:** Simona Imperio, Radames Bionda, Ramona Viterbi, Antonello Provenzale

**Affiliations:** 1 Institute of Atmospheric Sciences and Climate, CNR, Torino, Italy; 2 Ente di Gestione Delle Aree Protette dell’Ossola, Varzo, Italy; 3 Gran Paradiso National Park, Torino, Italy; University of Western Ontario, Canada

## Abstract

Alpine grouses are particularly vulnerable to climate change due to their adaptation to extreme conditions and to their relict distributions in the Alps where global warming has been particularly marked in the last half century. Grouses are also currently threatened by habitat modification and human disturbance, and an assessment of the impact of multiple stressors is needed to predict the fate of Alpine populations of these birds in the next decades. We estimated the effect of climate change and human disturbance on a rock ptarmigan population living in the western Italian Alps by combining an empirical population modelling approach and stochastic simulations of the population dynamics under the a1B climate scenario and two different disturbance scenarios, represented by the development of a ski resort, through 2050.The early appearance of snow-free ground in the previous spring had a favorable effect on the rock ptarmigan population, probably through a higher reproductive success. On the contrary, delayed snowfall in autumn had a negative effect possibly due to a mismatch in time to molt to white winter plumage which increases predation risk. The regional climate model PROTHEUS does not foresee any significant change in snowmelt date in the study area, while the start date of continuous snow cover is expected to be significantly delayed. The net effect in the stochastic projections is a more or less pronounced (depending on the model used) decline in the studied population. The addition of extra-mortality due to collision with ski-lift wires led the population to fatal consequences in most projections. Should these results be confirmed by larger studies the conservation of Alpine populations would deserve more attention. To counterbalance the effects of climate change, the reduction of all causes of death should be pursued, through a strict preservation of the habitats in the present area of occurrence.

## Introduction

Wildlife species living in mountain habitats are particularly vulnerable to environmental and climate change due to their adaptation to extreme and very specific conditions [1]. Global warming has been particularly marked in high-mountain areas in the last half century [2], with changes in snow amounts [3], climate-induced vegetational shifts [4], and subsequent cascade effects on animal species. This process is doomed to worsen if increases in temperature conform to recent climate models. 

Among homeotherms, birds are expected to respond rapidly to climate change both spatially, owing to their mobility (with distributional shifts as a result [5]), and temporally for the relative short generation time. For example, it has been estimated that, based on expected increasing temperatures during the breeding season, the potential habitat of rock ptarmigan *Lagopus muta* in Switzerland will decrease drastically by between one-quarter and two-thirds by 2070 [6]. Similar contractions of suitable habitats due to climate change are expected for other grouse species [7,8] Following a population dynamics approach, Wang et al. [9] suggested that future warming will accelerate the decline of white-tailed ptarmigan *Lagopus leucurus* abundance in the Rocky Mountain National Park, Colorado, USA.

Alpine grouses provide a good example of vulnerability to climate change as they are arctic species that occur as isolated glacial relict populations in the Alps. Rock ptarmigan is likely the grouse species which is most exposed to environmental changes as its breeding territories are located at the highest elevations above the timberline. The European Bird Directive (Council Directive 2009/147/EC) lists the Alpine rock ptarmigan *L. muta helvetica* in Annex 1, emphasising the need for special conservation measures for the preservation of this sub-species and its habitat. The Italian population of rock ptarmigan has been estimated as 5,000-8,000 pairs [10] with a constant decline of about 30% in the last decade, and it has been recently classified as Vulnerable in the Red List of Italian birds [11]. In contrast to sub-arctic and arctic populations, the ecology of alpine populations is poorly known [12] due also to the difficult environmental conditions one has to face to contact the individuals. 

The analysis of population density fluctuations is a key instrument for estimating the effects of limiting factors on population dynamics [13]. Population regulation by intrinsic mechanisms can be recognized when the population growth rate has a negative density dependence [14], although the effects of measurement errors have to be taken into account as they can spuriously amplify the effects of density dependence [15,16]. A spurious detection of a negative relationship between population growth rate and population size could then lead to the overestimation of the ability of a population to recover from decline through a feedback mechanism. Accounting for uncertainty in population size is, thus, especially important for the conservation of threatened species. 

Extrinsic factors, such as environmental stressors, can also be important determinants of population growth rate [17]. Climatic conditions can affect rock ptarmigan populations especially during the breeding season which is recognized as a key period for the population dynamics of this species. Peaks in June temperature are followed by peaks in rock ptarmigan populations in Scotland [18]. Novoa et al. [19] found a positive correlation between an early appearance of snow-free ground and reproductive success in the Pyrenees and also a negative effect of rainfall after hatching. On the contrary, Revermann et al. [6] found that suitable habitat for rock ptarmigans in Switzerland is associated with cold and wet July conditions, probably due to the inability of the species to dissipate body heat in the summer. Other periods could also be critical for the species, in particular those associated with autumn and spring molts when the match with the appearance/disappearance of snow cover is presumably crucial for the success of anti-predator strategies. 

Like other grouses, rock ptarmigans are also currently threatened by habitat modification and human disturbance, in particular by winter sports [20–22], but this aspect was never taken into account in the expected distribution estimates and projections of grouse populations (but see the simulations of Moss [23] for the Scottish population of capercaillie *Tetrao urogallus*, where the mortality of birds flying into forest fences was also considered). Watson and Moss [24] found 144 rock ptarmigans dead as a result of collisions with ski-lift wires in the period 1971-1996 in two areas of the Cairngorms massif (Scotland) and calculated a mean of 1.10 and 0.19 recorded deaths per km of wire per year, but observations of collisions revealed that the actual number of deaths was about five times higher. Dead birds recovered under ski-lift wires are in fact only a fraction of the actual victims of wire strike due to search bias, scavenger activity [25], and to some individuals flying away from the wire after the collision and dying far from it [26]. Bevanger and Brøseth [25] found a similar collision rate for ptarmigans on power lines in Norway (on average 5.3 birds km^-1^ per year). Surveys conducted in 252 French ski resorts [27] confirmed the vulnerability of grouse species to wire-strike mortality.

Both climate change and human disturbance can, thus, have a significant impact on rock ptarmigan populations, and an assessment of their contribution is needed if one wants to predict the fate of these Alpine populations in the next decades.

The aims of the present study are: 1) to detect the key factors driving the fluctuations of a population of rock ptarmigans living in the western Italian Alps without human disturbance, and 2) to predict the response of this population to expected climate change and to other possible disturbance sources, such as the development of a ski area.

## Methods

### Study species

Rock ptarmigans are widely distributed in the arctic, sub-arctic, and alpine environments of Europe, North America, and Asia with a global population estimated to number over 8,000,000 individuals [28]. The alpine population, on the southernmost edge of the distribution range, is relatively small (about 40.000 pairs) and declining by suffering from detrimental effects of human activities [29]. The Alpine rock ptarmigan is a small grouse with an adult body mass of about 385–520 g [29], which occurs in flocks in autumn and winter, and is territorial when breeding [12]. The breeding season is between the second half of May and June, and chicks hatch in the first half of July [10] (mean number of young per hen in summer: 0.6-5 [28]). The pre-nuptial molt starts from the end of April and it is complete in May or June, while the autumn molt starts from the end of September until complete white plumage is attained between November and December [10]. 

### Study area

The study was conducted in the Alpe Veglia and Alpe Devero Natural Park (46°19′N, 8°14′E) represented by large mountain basins of glacial origins and surrounded by the summits of the western Lepontine Alps (1600-3553 m asl). The breeding habitat of the species is mainly located at elevations ranging from 2100 m to 2700 m asl with the lower parts dominated by scattered woods of larch *Larix decidua* and a rich understory of bilberry (*Vaccinium myrtillus, V. uliginosum*) and alpenrose *Rhododendron ferrugineum*, while the upper parts of the habitat are characterized by meadows (Poaceae and Cyperaceae), heaths with alpine azalea *Loiseleuria procumbens*, and screes with sparse alpine vegetation (*Silene acaulis*, *Chrysanthemum alpinum*, *Myosotis alpestris*, *Ranunculus glacialis*, *Aster alpinus* etc.). 

During the study period at 2200 m asl, average monthly temperatures were -6.4°C in January and 10.1 in August, while mean annual precipitation was 1180 mm. At the same site, the start date of continuous snow cover was between October 10^th^ and November 25^th^ (median November 2^nd^), while the end date was between May 13^th^ and June 25^th^ (median June 7^th^).

About 4 km from the census area, a small ski resort with a total ski-lift length of less than 2 km was built in the ‘70s, is still in operation, and is only marginally occupying the ptarmigan habitat. Hunting is forbidden in the Park (since 1992), but it is allowed in the neighbouring buffer zone and hunting districts where no rock ptarmigans were shot during the study period and the black grouse is the main quarry species. 

### Meteorological data

Daily temperature (minimum and maximum), precipitation, and snow depth were collected by a meteorological station at 2200 m asl placed on the dam of the Lake Vannino about 6 km away from the census area. Meteorological data can be requested to Enel UBH Piemonte (but see Table S2 for the aggregated quantities used in this study).

For subsequent analyses, we computed the daily mean temperature (as the average between minimum and maximum temperature), the start/end ordinal date of continuous snow cover (start date: number of days from 1^st^ September; end date: number of days from 1^st^ May), and the total number of days with continuous snow cover in each winter. We then averaged the mean temperature, precipitation, and snow depth across 6 critical phases in the life cycle of the species: 1. spring molt and breeding before egg-laying (April-May), 2. egg-laying and brooding (June), 3. hatching and rearing (July), 4. autumn molt (September-October), 5. first part of winter (November-December), and 6. second part of winter (January-March). 

Snow depth and mean temperature have a significant negative correlation within each phase, except for the first (r=-0.25, p=0.25) and second part of winter (r=-0.15, p=0.50). Precipitation and mean temperature are negatively correlated only in June (r=-0.47, p=0.03) and July (r=-0.51, p=0.01), and snow depth and precipitation are correlated only in autumn (r=0.68, p=0.009) and in the first part of winter (r=0.72, p<0.001). In most cases, snow depth is significantly correlated between contiguous phases (e.g. between April-May and June: r=0.70, p=0.0002), and, in some cases, precipitation is significantly correlated with the snow depth of the following phase (e.g. in the first and second part of winter, respectively: r=0.77, p<0.0001).

The number of days with snow cover on the ground is significantly correlated with the start date of snow cover (r=-0.82, p<0.0001), snowmelt date of the following spring (r=0.73, p<0.001), snow depth (r=0.51, p=0.04) and mean temperature in autumn (r=-0.56, p=0.02), snow depth in the second part of winter (r=0.61, p=0.01) and in the following spring (r=0.69, p=0.002). The start date of snow cover is significantly correlated with snow depth (r=-0.58, p=0.01) and mean temperature in autumn (r=0.47, p=0.05), and with snow depth (r=-0.48, p=0.05) and precipitation in the first part of winter (r=-0.59, p=0.01); snowmelt date is significantly correlated with snow depth (r=0.81, p<0.0001) and mean temperature in spring (r=-0.66, p=0.003), and snow depth in June (r=0.81, p<0.0001). None of the climatic variables (including start/end date and days of snow cover) were significantly auto-correlated at lag 1 (that is, there is no correlation between subsequent years).

Since the values of snow depth in the different phases are always linearly correlated with the temperature or the duration of the snow cover period, in the subsequent analyses we did not include snow depth. To further reduce the number of independent variables, we considered precipitation only in the brooding (June) and hatching (July) periods, as these are likely to affect the reproductive success [19]. 

### Counts of calling cocks

For the census of rock ptarmigan cocks, we used the sampling method suggested by Bossert [30] and Léonard [31] based on the count of calling cocks in the period preceding egg-laying by hens. The area potentially occupied by the study population extends over 60 km^2^ and is too large to be surveyed exhaustively. We, therefore, decided to focus on a smaller sample area taking into account the suitability for the species, the representativeness of the altitudinal range occupied by the species in spring, and the accessibility by the operators. The chosen site lies in the Devero Valley and has an area of 2.66 km^2^.

Censuses were conducted each year from 1996 to 2012 between the end of May and the first half of June (depending on the snow cover and the accessibility of the area) when the calling activities of rock ptarmigan cocks peak [32]. Counts were only conducted during calm and dry weather conditions from 7 vantage points at an average distance of about 500 m (range: 360-688 m) from each other.

Observation points were placed in the sample area to obtain a comprehensive covering (visual and acoustic) of the area considering that territorial cocks can be heard up to 1 km away [32]. Operators reached the assigned points at the latest half an hour before dawn, they recorded the number and the time of calls and marked the positions of calling cocks on a 1:25000 map. Finally, the data collected by neighbouring operators were compared to avoid double counts. 

The permission for field work in the park was issued by Ente di Gestione delle Aree Protette dell’Ossola.

### Data filtering

By dividing the total number of counted cocks in each year by the monitored area, we estimated the number of calling cocks per km^2^. As rock ptarmigan is a monogamous species, each territorial male is generally paired with a single female [33]. Thus, the number of breeding pairs can be estimated from the observed spring cock density. However, since density estimates were potentially affected by observation errors, we used a state-space model (SSM) to filter the original raw data and disentangle sampling error from environmental variability. This was necessary to avoid biases in the detection of a negative relationship between population growth rate and population size [15,16].

SSMs, when used for analyzing time series of population abundances, allow the joint estimation of the likelihood of the amount of observation error along with the amount of process noise. In particular, we adopted a linear SSM with a Kalman filter approach assuming a Gompertz density dependence, which represents environmental stochasticity as a lognormal stochastic process and includes lognormal observation errors [34]. For parameter estimation, we used the PROC MIXED of SAS 9.2 (SAS Institute Inc., Cary, NC) following the procedure step by step described in the Appendix B of the paper by Dennis et al. [34].

### Estimate of climatic effects

To analyze population fluctuations, we estimated the annual growth rate, *R*_*t*_=*ln*(*Nt*/*N*_*t*−1_), where *N*_*t*_ is the Kalman-filtered population density on year *t*. The estimated annual growth rate was then used as the dependent variable in a series of generalized linear models (GLM) used to test a modified version of the Gompertz population model [35]:

$$R_{t}=a+b\ln N_{t-1}+\sum_{i=1}^{v}c_{i}V_{i}+\varepsilon$$ Eq.1

where *V*_*i*_ is one of the meteorological variables described before (related to the phases spanning from spring of the year *t-1* to spring of the year *t*) and ε is a Gaussian and temporally uncorrelated random variable. We also tested models without density dependence (that is, suppressing the second term on the right hand side of Eq. 1), or with delayed density dependence (that is, replacing the term *N*_*t-1*_ with *N*_*t-2*_ in Eq. 1). We tested all linear combinations of variables with the limitation of using a number of independent variables that did not exceed a ratio of 1:5 to the number of available data points (that is, 3 variables at most) while avoiding models containing two variables that were significantly cross-correlated in order to reduce problems with parameter estimations [36]. 

The most appropriate models were selected using the Aikake’s Information Criterion with finite sample correction (AICc, [37]). 

### Population projections

To estimate future population trends, we forced the best-performing models with the time series of meteorological variables (temperature, precipitation, and snow depth from which we derived the start date of a continuous snow cover, snowmelt date, and the total number of days with snow cover) generated by the PROTHEUS regional climate model [38,39] for the A1B scenario in the period 2013-2050. The A1B is a standard greenhouse gas emission scenario used for climate projections in future decades and adopted in the IPPC AR4 report [40]. The A1B scenario makes the hypothesis of a balance between fossil and non-fossil energy sources where balanced is defined as not relying too heavily on one particular energy source, and it works on the assumption that similar improvement rates apply to all energy supplies and end-use technologies. PROTHEUS is a state-of-the-art coupled ocean-atmosphere regional climate model developed by ENEA and ICTP for the Mediterranean region based on the RegCM3 atmospheric model and the MITgcm ocean model. The results of the PROTHEUS scenario simulations can be freely downloaded as indicated in the Appendix S1. The model configuration has a uniform grid spacing of 30 km; for the present study, we used the output of the model for the grid cell including the study area.

Snow depth is provided in mm of snow water equivalent; for simplicity we assumed a constant snow density within the year and throughout the simulation period. To standardize the model’s meteorological variables for subsequent analyses, all PROTHEUS time series were scaled to have the mean and variance of the observed Vannino series in the period 1991-2010. 

When population density is very low, phenomena such as inbreeding, Allee effect, and demographic stochasticity can lead populations to extinction. To overcome these problems that imply a dynamics which is not described by the population model used here, we used a quasi-extinction threshold [41]: if the population density drops below this threshold, it is considered extinct or on the inevitable way to extinction. Given the extension of the area potentially occupied by the population, we chose a threshold of 0.1 ind/km^2^, corresponding to 5-6 cocks in the study area.

Besides climate change, we simulated the effect of the development of ski resorts (with 2 levels of development) as an example of human disturbance. To this end, we inserted an extra-mortality term in Eq. 1, called *d*:

$$N_{t}=N_{t-1}e^{(a+b\ln N_{t-1}+\sum_{i=1}^{v}c_{i}V_{i}+\varepsilon )}-(d\frac{N_{t-1}}{11.8}+0.02)$$ Eq.2

There is little reason to believe that mortality caused by collisions with wires should be compensatory [25]. A non-natural source of mortality, as a matter of fact, is more likely to be additive in low-density or declining populations [42]. Moreover, additivity is more likely if the supplementary mortality overlaps with or follows periods of natural mortality [43]. Given that natural mortality of rock ptarmigans in the Alps occurs mainly between June and October [44], while wire-strike mortality peaks in winter [24,25], it is reasonable to approximate this human-induced source of mortality as an additive contribution to natural mortality. Based on the death data due to collision with ski-lift wires collected by Watson and Moss [24] (for simplicity, we imagined a ratio of 1 km of wire per 3 km^2^) and a correction factor equal to 5 for the search bias as resulted by direct observations [24], *d* was put equal to 1.83 individuals per km^2^ for a highly developed area (i.e. with car parks and other tourist facilities) and to 0.32 birds per km^2^ for a less developed area. We related this mortality rate to the average density recorded in one of the areas surveyed by Watson and Moss [24] in the period 1967-1996 (area “E”: 11.8 [S.E. 0.97] birds/km^2^), as Bevanger and Brøseth [25] found the collision rate to be linearly related to density. Hence, the supplementary mortality due to wire strike resulted 0.16 *N*_*t-1*_ and 0.03 *N*_*t-1*_ for the highly and less developed ski resorts, respectively. To avoid non-integer mortality rates at low density, we added a minimum constant term of 1 dead bird per year in the whole potential occupied area (1/50 km^2^).

The values indicated above are underestimates of the true mortality levels caused by tourist infrastructure development and by the subsequent increase in touristic influx. Such an increase can affect rock ptarmigans both directly (by human disturbance in winter and summer) and indirectly, due to the possible increase in the number of nest predators such as carrion crow *Corvus corone* [24] and red fox *Vulpes vulpes*. Collision with snow fences is another mortality source in ski areas [24]. 

The results of the simulation of the joint effects of climate and human disturbance was then compared with a simulation of a population simply driven by density dependence and human disturbance (without climatic effects) and carried out by fitting a simple Gompertz population model (i.e., removing the third term of the right hand side of Eq. 1) to the time series of population densities.

All population projections have been performed with the R statistical package, ver. 2.13.2 [45].

## Results

### Spring densities

**Figure 1:**
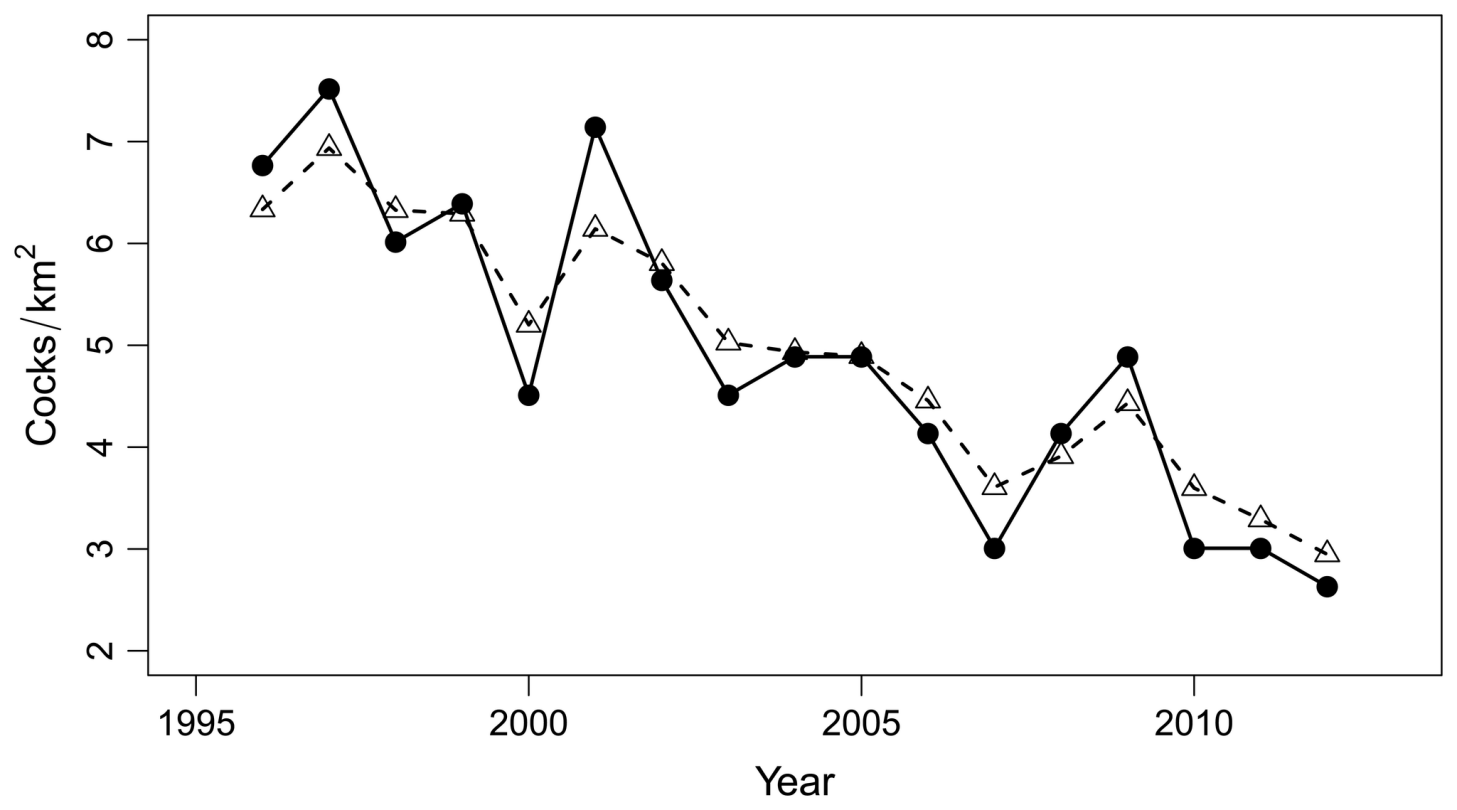
Time series of population densities. Observed spring cock densities in the period 1996-2012 (dots and solid line) and estimated breeding pairs densities from the fitted Gompertz state-space model (triangles and dotted line).

Across the 16 years of this study, the density of rock ptarmigan cocks varied from 2.63 calling cocks per km^2^ in 2012 to 7.52 cocks/km^2^ in 1997 (Figure 1 and Table S1) with a significant decreasing trend (R^2^=0.76, p<0.0001) leading to the average loss of 0.26 cocks/km^2^ per year. The population growth rate time series, on the contrary, shows no trend (R^2^=0.01, p=0.73).

The state space model yielded a series that was filtered by measurement errors (Figure 1) with an estimated variance of real population densities time-series (σ^2^) of 0.016 and a variance of the observation error (τ^2^) of 0.023 (see 34 for further information).

### Factors affecting population fluctuations

The results of the GLMs are reported in Table 1, which lists the best 10 models (in the AICc sense). All models reported here are highly significant (p<0.01), and the explained variance is between 69% and 83%.

**Table 1:** Selection of the models for the growth rate of rock ptarmigans in the Veglia-Devero Natural Park.

Model	Intercept	lnN_t-1_	lnN_t-2_	SE_t-1_	SS_t-1_	SP_t_	T(July)_t-1_	P(July)_t-1_	T(Jan-Mar)_t_	T(Apr-May)_t_	var.	R^2^	AICc
M1	-0.07±0.04			**-0.19±0.04**	**-0.18±0.04**						2	0.78	-50.53
M2	0.34±0.24		-0.25±0.14	**-0.19±0.04**	**-0.19±0.04**						3	0.83	-50.20
M3	-0.07±0.04			**-0.19±0.04**	**-0.18±0.04**			0.05±0.03			3	0.82	-49.28
M4	-0.07±0.04			**-0.19±0.04**	**-0.17±0.04**		-0.05±0.04				3	0.81	-48.51
M5	-0.07±0.04			**-0.20±0.04**	**-0.18±0.04**				-0.03±0.04		3	0.79	-47.28
M6	0.08±0.26	-0.10±0.16		**-0.18±0.04**	**-0.17±0.04**						3	0.78	-46.98
M7	-0.07±0.04			**-0.19±0.04**	**-0.18±0.04**					-0.01±0.04	3	0.78	-46.51
M8	0.42±0.25	-0.30±0.15		**-0.14±0.04**		**0.16±0.04**					3	0.77	-46.34
M9	-0.07±0.04			**-0.17±0.05**		**0.16±0.05**					2	0.69	-45.79
M10	-0.08±0.04			**-0.18±0.04**		**0.19±0.05**				0.08±0.05	3	0.75	-45.02

Model ID, parameter estimates ± standard error for intercept and selected variables (lnN_t-1_: log-normal density at time *t-1*; lnN_t-2_: log-normal density at time *t-2*; SE: snow cover end date; SS: snow cover start date; SP: length of snow cover period; T: mean temperature; P: precipitation), number of variables included in the model, coefficient of determination (R^2^), and AICc are given for each model. Significant factors are in bold.

**Figure 2:**
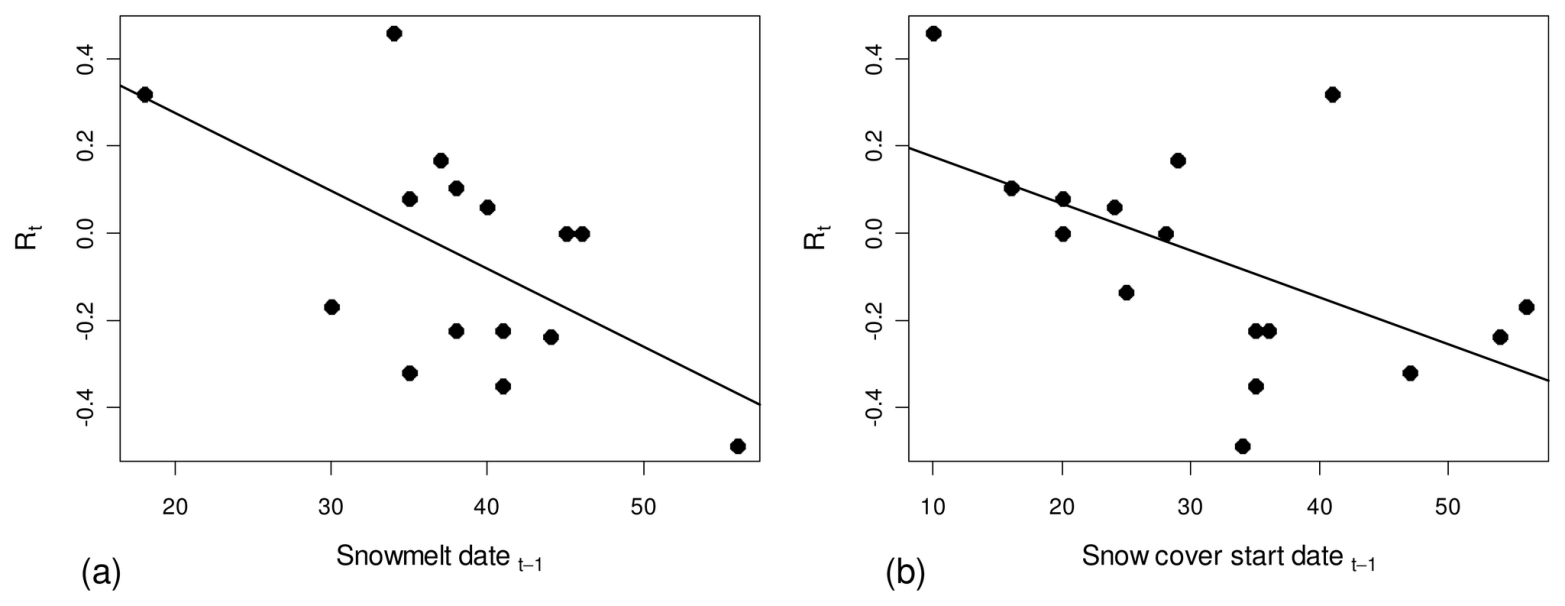
Climatic effects on rock ptarmigan population dynamics. Relationship between the population growth rate and snowmelt date at time *t-1* (a) (days from 1^st^ May) and start date of a continuous snow cover at time *t-1* (b) (days from 1^st^ October).

Snowmelt date at time *t-1* and the start date of snow cover in the following autumn were the most significant effects explaining 78% of the variance. Generally speaking, the early appearance of snow-free ground in the previous spring had a favorable effect on the rock ptarmigan population, while late snowfalls in autumn had a negative effect (Figure 2). The same result was obtained with the positive effect of the length of snow cover period, which is negatively correlated with the start date of snow cover. Other non significant climatic effects are represented by the temperature and rainfall in July at time *t-1* and the temperature in the second part of winter and in spring at time *t*. 

Delayed density dependence appears as a non-significant effect in two models, including the second best model, while direct density dependence is present in 6 out of the 15 models and resulted in a significant effect in half of them.

### Rock ptarmigan response to climate change and human disturbance

**Figure 3:**
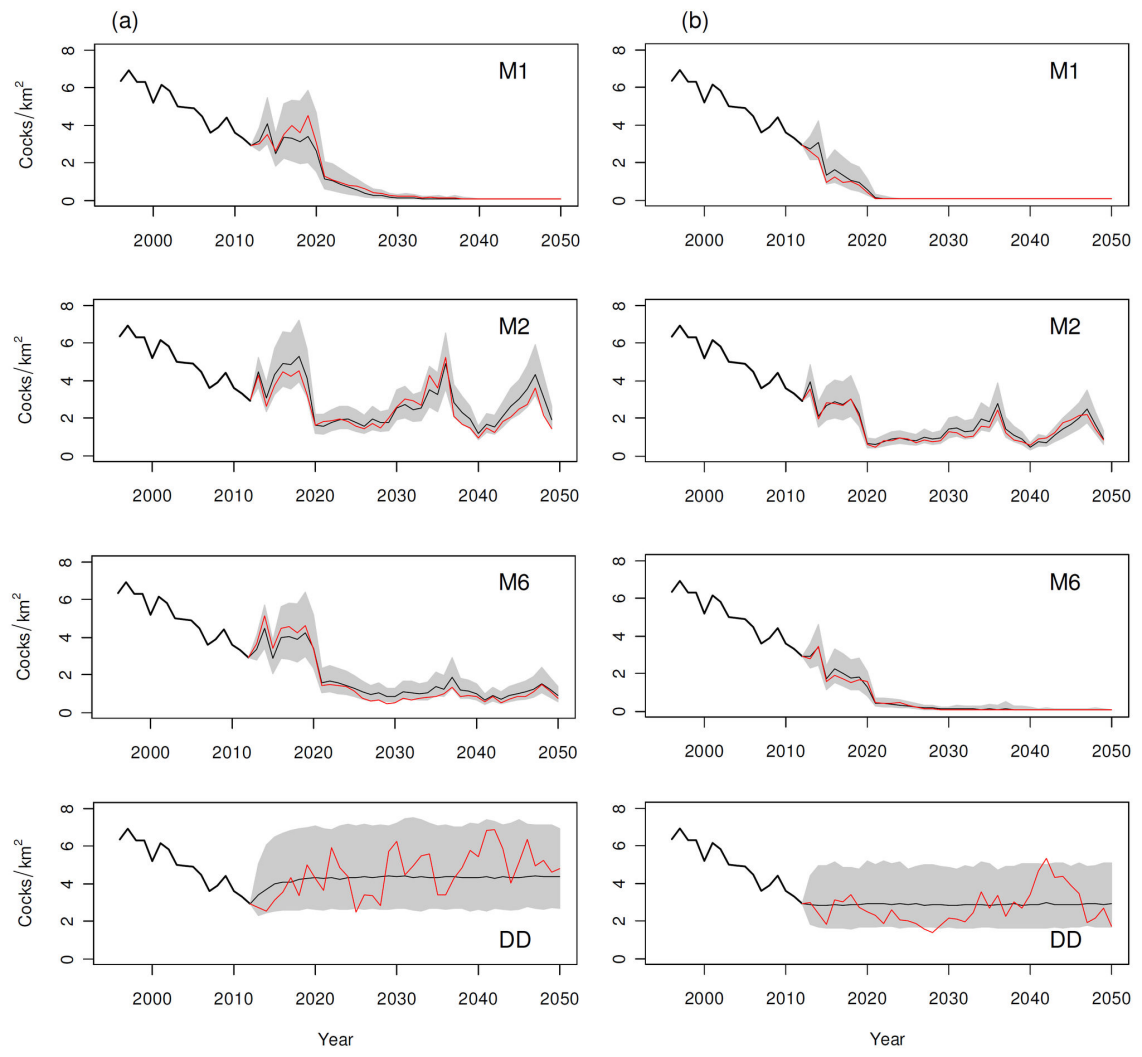
Population projections of rock ptarmigans for the period 2013-2050. (a) Projections performed using populations models M1, M2, and M6 (see Table 1) or a simple Gompertz density dependence model (DD) and the meteorological variables generated by the PROTHEUS model for the A1B scenario; (b) simulations of the joint effect of climate change and human disturbance using the same models as before and an extra-mortality term due to wire collision in a highly developed ski resort (0.16 *N*_*t-1*_ + 0.02 individuals/km^2^ per year). Thick line: estimated breeding pairs densities (cocks/km^2^); thin line: 50% percentile, shaded area: 5–95% percentiles of the 1000 runs; the red line represents one random realization.

All the population projections performed using the models described above and the meteorological variables produced by the PROTHEUS regional model for the A1B scenario predicted a more or less pronounced decline (Figure 3a). The best performing population models (Table 1) include a negative effect of a late snowmelt date and, above all, a negative effect of the start date of continuous snow cover. The spring snowmelt is expected to not change significantly in the coming decades in the study area (the trend in snowmelt date, as estimated from the snow depth output of the PROTHEUS model, is -0.06 days/yr with p=0.56 for the period 1990-2050). The start date of continuous snow cover is expected to be significantly delayed (the trend in the start date of snow cover, as estimated from the snow depth output of the PROTHEUS model, is 0.26 days/yr with p=0.005 for the period 1990-2050). As a consequence, simulations carried out with the model M1 (see Table 1) predicted the rock ptarmigan population to continue declining with a similar trend as the current one and become extinct with a 50% probability by 2033 and 95% by 2040. Simulations carried out with models M3, M4, and M5, not shown here, produced similar results. Introducing a delayed density dependence term (model M2, see Table 1) prevented the population from extinction with only a slight decline (average of the 50% percentile of each year in the period 2041-2050: 2.50 [S.E. 0.32] cocks/km^2^). Model M6 (including a direct density dependence term) yielded intermediate results between the models M1 and M2 by predicting a steady decline and then a stabilization on low densities (average of the 50% percentile in the period 2041-2050: 1.01 [S.E. 0.08] cocks/km^2^).

The addition of an extra-mortality term due to collision with ski-lift wires, in the scenario with limited development of ski areas (0.03 *N*_*t-1*_ + 0.02 individuals/km^2^ per year), produced results that were not very different from the simulation of a population driven only by climate (and density dependence, where present). Projections performed using model M1 predicted an anticipation of the extinction time by about 4-9 years compared to the undisturbed population, while models including density dependence foresaw a further lowering of the population densities (average of the 50% percentile in the period 2041-2050: 2.16 [S.E. 0.29] cocks/km^2^ for model M2, 0.61 [S.E. 0.05] cocks/km^2^ for model M6).

The simulation of a disturbance caused by highly developed ski areas (0.16 *N*_*t-1*_ + 0.02 extra-deaths/km^2^ per year), on the contrary, led to fatal consequences in almost all the projections (Figure 3b). Using the model M1, the rock ptarmigan population is expected to become extinct within 10 years (with a probability of 50% by 2022 and 95% by 2024). Projections carried out with the model M2 predicted a probability of 1% for extinction and an average of the 50% percentile during the period 2041-2050 of 1.32 [S.E. 0.20] cocks/km^2^. Using the model M6, we expect the population to become extinct with a 50% probability by 2038 and 95% by 2050.

Finally, projections carried out with a simple Gompertz population model (without climatic effects) showed no trend in the undisturbed population (average of the 50% percentile in the period 2041-2050: 4.36 [S.E. 0.01] cocks/km^2^) and only a slight decline in the population subjected to human disturbance (average of the 50% percentile in the period 2041-2050: 3.96 [S.E. 0.02] cocks/km^2^ with a less developed ski area, 3.03 [S.E. 0.01] cocks/km^2^ with a highly developed ski area) (Figure 3).

## Discussion

Spring cock densities of rock ptarmigans in the Veglia-Devero Natural Parks are similar to those found in other Alpine regions (1.5 to 6.7 territorial cocks per km^2^ in the Austrian Alps [46]; 4-5 cocks/km^2^ in Switzerland [47]; between 1.1 and 4.8 in Alto-Adige, Italy [48]), and they display a similar decline (on average, about 30% in 10 years in Italy [11]). Understanding the causes of this decrease is essential for predicting future trends of this population, as well as populations living in a similar environment, and to properly address conservation measures.

Climate represented the main driver of growth rate in this population. In particular, the snowmelt date during the spring of the first of the two censuses from which the growth rate is calculated, and the start date of snow cover in the autumn between the two counts explain 78% of the variance. These two variables are not cross-correlated and represent distinct effects in two definite phases of the life cycle of the species. 

The early appearance of snow-free ground during the spring of the first census is associated with the early timing of breeding and a higher reproductive success [19]. This effect could be mediated by the early plant growth and hence the foraging conditions of the hen before laying [49]. In this context, rock ptarmigans should be favoured by global warming, as snowmelt is expected to occur earlier in the next decades. However, since the rock ptarmigan is a species adapted to the harsh conditions of the arctic and high mountain areas, higher temperatures in summer could be unfavourable to the populations. Revermann et al. [6] found, in fact, that regions with the lowest temperatures (below 10–12 C°) and receiving higher amounts of precipitation in July represent more suitable habitats for the Alpine rock ptarmigan. The temperature constraint can be explained by the “heat dissipation theory” [50] according to which endothermic animals are limited by the ability to dissipate body heat rather than by the competition for a limited energy supply. Hence, rock ptarmigans are favoured by an early snowmelt but not by high summer temperatures that may cause hyperthermia problems despite the higher availability of food supply in warmer years. The negative effect of high summer temperatures was also detected in our analysis, albeit the signal was not statistically significant.

The starting date of continuous snow cover in the autumn between the two censuses, on the contrary, affects the mortality of both adults and first-winter birds. Starting from the end of September, rock ptarmigans complete their molt (as a supplemental post-nuptial molt) to an almost completely white winter plumage [10]. This snow camouflage is necessary since these birds live year-round in open areas where they are particularly vulnerable to predation. The white plumage on the bare ground, on the other hand, represents one of the most conspicuous plumages known in birds and increases predation risk [51]. Considering that predation from raptors, particularly the golden eagle *Aquila chrysaetos* in Italian Alps, is the main form of adult mortality [44,52,53], we can understand the importance of the match between molt and the start of snowfalls. In the French Alps, the female rock ptarmigan survival rate is lowest in October [44], and autumn is the season when willow grouses *Lagopus lagopus* in Sweden suffer the higher mortality due to natural predation [54]. These results point towards a higher vulnerability in this time of the year that could be enhanced by late snowfalls. Winter molt, in fact, is triggered by changes in the photoperiod [55] even though temperature seems to also have some importance [56]. However, it can be difficult for rock ptarmigans to forecast such a highly variable meteorological event exactly (start date of a continuous snow cover spanned over 46 days in the study period).

One should expect to observe the same vulnerability in spring, during the transition from the white plumage to the breeding one, but there are at least two reasons why this did not occur. First, pre-nuptial molt is incomplete turning upper parts to a greyish colour while wings and under parts remain white [10]. This favors the camouflage during snowmelt and is helped by the cryptic feeding behavior at the border zone between snow and bare ground [57]. Secondly, eagles have a high availability of prey in the spring (e.g. Alpine marmots *Marmota marmota*, juvenile ungulates), thus, predation pressure on ptarmigans is reduced during this season. In a different situation in arctic North America with a predator specialized on rock ptarmigan (gyrfalcon *Falco rusticolus*) and the lack of alternative prey, spring is indeed the season with the highest mortality due to predation [51].

It has to be noted that the population of golden eagle in the western Italian Alps is increasing [58], as well as other potential predators (other raptors, red fox) due to a decrease in human persecution and an increase in the carrying capacity of the mountain habitat for these species. However, unlike arctic areas, none of the predators present in the area is specialized on rock ptarmigan. Hence, we believe this occurrence could have enhanced the effect of higher vulnerability in autumn, rather than act as an important driver of grouse population itself. 

Besides climatic effects, social and trophic interactions that cause density dependence represent another, yet less important, driver of population fluctuations. Both direct and delayed density dependence entered the best-performing models mostly as non-significant factors. However, it can be difficult to detect density dependence in a population that is strongly affected by external factors [59], such as snow cover in this case. In addition, the limited extension of our study area does not allow unambiguous identification of the form of density dependence. 

Despite the clarity of the outcomes provided by the empirical population models considered in this study, generalization of the results to the whole Alpine range should be made with caution due to the complexity of habitat-population relationships and the possible influence of site-dependent factors. 

Stochastic projections of population dynamics using the output of the PROTHEUS regional climate model predicted a worrying decline in the rock ptarmigan population under study. While the advance of snowmelt date in spring should favor breeding success, the prevailing effect on the population growth rate seems to be a negative effect of the predicted delays in the start date of a continuous snow cover in autumn. Models without density dependence suggested a steady decline that will inevitably lead to population extinction; however, it is unlikely that some form of density dependence, even though weak, will not operate on the population dynamics of ptarmigans (a priori, density dependence can be expected in real populations with long-term persistence [14]). Models with density dependence, in fact, showed a clear decline of the population which will, however, stabilize at very low densities. 

Rock ptarmigans already live at the highest elevations in the Alps, and it is difficult for them to move further uphill. One possible adaptation to climate change could be a shift in the timing of molt, to be achieved through an evolutionary change in the physiological responses to photoperiod. However, the response to this selection may be slow [60]. Moreover, a genetic shift in thermal tolerance as an adaptation to global warming has never been demonstrated in animals [61], and the intolerance to high summer temperatures may, in the long run, become a serious constraint to the survival of rock ptarmigans in the area studied. 

The effect of supplementary mortality due to the simulated development of a ski resort raises further concerns for the preservation of Alpine populations of rock ptarmigan. The study population, already damaged by the changing duration of snow cover, could not tolerate high levels of wire strike mortality without incurring a severe decline or even local extinction in a few decades. The projections provided by a simple Gompertz population model revealed that a supplementary mortality source, such as the presence of ski-lifts, in absence of climate change is less harmful. Hence, it is the concomitant effects of the two stressors that may have critical and fatal consequences on the population. 

As already mentioned, values used in the simulations are underestimates of the true mortality levels caused by touristic development; therefore, the results presented in this study should be considered as conservative estimates of future population levels. In principle, ski resorts should be distributed in the whole study area to have lethal effects on the overall population. However, in winter, birds can move in flocks across larger areas than in spring and could, thus, die on wires that are far from their territories [24]. In any case, it is clear that any kind of human disturbance with similar mortality effects on ptarmigans (e. g. construction of power lines, opening of roads, illegal hunting) will result in a serious detriment to a population that is already compromised by climate change. In addition to this, we have to consider the predicted reduction in the suitable habitat for rock ptarmigans in the Alps due to global warming [6].

Outside protected areas, hunting is another source of mortality for the species. Hunting bags during the last 16 years in Piedmont underwent an even steeper decline than density estimates resulting from censuses: in the Verbanio-Cusio-Ossola Province (which includes the Veglia-Devero Park), shot ptarmigans decreased from an average of 18.2 [S.E. 3.26] per year in the period 1996-2000 to 5.67 [S.E. 1.57] specimens per year in the period 2006-2010 (excluding the years 2007-2008, when rock ptarmigan hunting was closed). During these years, bag size reached the hunting quota only in 1998 (data from VCO hunting district). Despite the fact that hunting takes place before the effect of natural winter mortality (even though the natural mortality of rock ptarmigans in the Alps seems to be high in summer and autumn [44]) and quotas are determined according to the results of spring censuses in each hunting district, it has been shown that harvest is only weakly compensatory in ptarmigans [62] and declining populations could not tolerate an additional removal of individuals. In Italy, rock ptarmigan hunting is allowed from October 1^st^ to November 30^th^, a period during which, in the last years, ptarmigans are particularly vulnerable due to late snowfalls as revealed by the present study. 

Should the results of the present work be confirmed by larger studies, the conservation of Alpine populations would deserve more attention. To counterbalance the effects of climate change, the reduction of all causes of death should be pursued. Hence, conservation strategies should be addressed for a strict preservation of the habitats in the present area of occurrence, and a carefully planned or closing of hunting activity is required to avoid local extinctions of this vulnerable sub-species.

## Supporting Information

Appendix S1 **Instructions for downloading the results of the PROTHEUS scenario simulations.**
(DOC)

Table S1 **Observed rock ptarmigan cocks for the spring counts in the Devero Valley.**
(DOC)

Table S2 **Meteorological variables entering the best performing models.**
(DOC)
