# Supplementary material for: Climate Change and Human Disturbance Can Lead to Local Extinction of Alpine Rock Ptarmigan: New Insight from the Western Italian Alps
Source: PLoS One. 2013 Nov 19;8(11):e81598. doi: 10.1371/journal.pone.0081598 (PMC3834331; doi:10.1371/journal.pone.0081598)
Supplement: Appendix S1 — Instructions for downloading the results of the PROTHEUS scenario simulations. (DOC) [file pone.0081598.s001.doc]

**Appendix S1**

Numerical simulations for various climate scenarios can be freely downloaded from the website of the NextData Project, <http://www.nextdataproject.it/?q=content/model-data>.

The A1B scenario results are produced by simulations performed by ENEA using the PROTHEUS model, in particular, for the EH5OM_A1B run used here, see <http://utmea.enea.it:8080/thredds/catalog/CIRCE1/EH5OM_A1B/catalog.html>
