# Supplementary material for: Climate Change and Human Disturbance Can Lead to Local Extinction of Alpine Rock Ptarmigan: New Insight from the Western Italian Alps
Source: PLoS One. 2013 Nov 19;8(11):e81598. doi: 10.1371/journal.pone.0081598 (PMC3834331; doi:10.1371/journal.pone.0081598)
Supplement: Table S1 — Observed rock ptarmigan cocks for the spring counts in the Devero Valley. (DOC) [file pone.0081598.s002.doc]

**Table S1**. Observed rock ptarmigan cocks for the spring counts in the Devero Valley (Alpe Veglia and Alpe Devero Natural Park, Italy) and related density estimates.

| Year | N | d |
| --- | --- | --- |
| 1996 | 18 | 6.77 |
| 1997 | 20 | 7.52 |
| 1998 | 16 | 6.02 |
| 1999 | 17 | 6.39 |
| 2000 | 12 | 4.51 |
| 2001 | 19 | 7.14 |
| 2002 | 15 | 5.64 |
| 2003 | 12 | 4.51 |
| 2004 | 13 | 4.89 |
| 2005 | 13 | 4.89 |
| 2006 | 11 | 4.14 |
| 2007 | 8 | 3.01 |
| 2008 | 11 | 4.14 |
| 2009 | 13 | 4.89 |
| 2010 | 8 | 3.01 |
| 2011 | 8 | 3.01 |
| 2012 | 7 | 2.63 |
