# Supplementary material for: Climate Change and Human Disturbance Can Lead to Local Extinction of Alpine Rock Ptarmigan: New Insight from the Western Italian Alps
Source: PLoS One. 2013 Nov 19;8(11):e81598. doi: 10.1371/journal.pone.0081598 (PMC3834331; doi:10.1371/journal.pone.0081598)
Supplement: Table S2 — Meteorological variables entering the best performing models. (DOC) [file pone.0081598.s003.doc]

**Table S2**. Meteorological variables entering the best performing models (see Table 1); by courtesy of Enel UBH Piemonte.

| Year | Snowmelt date | Start date of snow cover | Length of snow cover period (days) | Mean Temp. Jan - Mar (°C) | Mean Temp. Apr - May (°C) | Mean Temp. July (°C) | Mean Prec. July (mm) |
| --- | --- | --- | --- | --- | --- | --- | --- |
| 1996 | 07 Jun | 16 Oct | 218 | -6.11 | 1.88 | 8.84 | 5.48 |
| 1997 | 07 Jun | 05 Nov | 234 | -2.89 | 1.42 | 8.56 | 3.52 |
| 1998 | 09 Jun | 24 Oct | 216 | -3.13 | 1.39 | 10.52 | 2.06 |
| 1999 | 10 Jun | 04 Nov | 229 | -5.01 | 2.33 | 10.23 | 2.39 |
| 2000 | 03 Jun | 10 Oct | 212 | -4.36 | 2.16 | 7.76 | 4.81 |
| 2001 | 13 Jun | 23 Nov | 246 | -4.71 | 1.66 | 10.06 | 6.74 |
| 2002 | 10 Jun | 04 Nov | 199 | -3.69 | 1.57 | 9.61 | 4.19 |
| 2003 | 04 Jun | 20 Oct | 212 | -5.84 | 1.98 | 11.82 | 3.52 |
| 2004 | 14 Jun | 28 Oct | 238 | -5.08 | 0.96 | 9.19 | 1.58 |
| 2005 | 30 May | 25 Nov | 214 | -6.14 | 1.93 | 10.10 | 1.55 |
| 2006 | 04 Jun | 16 Nov | 191 | -6.93 | 1.89 | 13.44 | 2.16 |
| 2007 | 18 May | 10 Nov | 183 | -2.86 | 4.03 | 9.58 | 2.97 |
| 2008 | 06 Jun | 29 Oct | 209 | -3.91 | 1.17 | 9.63 | 4.77 |
| 2009 | 25 Jun | 03 Nov | 239 | -6.56 | 2.70 | 9.92 | 1.94 |
| 2010 | 15 Jun | 20 Oct | 224 | -7.72 | 1.05 | 11.65 | 2.03 |
| 2011 | 13 May | 25 Oct | 205 | -4.77 | 4.16 | 7.94 | 7.42 |
